# Persistent Non-*albicans* Predominance, High Mortality, and Azole-Non-Susceptible *Candida tropicalis*: A 15-Year Pediatric Invasive Candidiasis Cohort in Southern Thailand

**DOI:** 10.3390/jof12090664

**Published:** 2026-09-03

**Authors:** Puttichart Khantee, Kochakorn Pinichkijpaisal, Mingkwan Yingkajorn, Therdpong Thongseiratch, Kamolwish Laoprasopwattana

**Affiliations:** 1 Department of Pediatrics, Faculty of Medicine, Prince of Songkla University, Hat Yai 90110, Thailand; kochakornping@gmail.com (K.P.); ttherd@gmail.com (T.T.); kamolwish@gmail.com (K.L.); 2 Clinical Microbiology Unit, Department of Pathology, Faculty of Medicine, Prince of Songkla University, Hat Yai 90110, Thailand; mingkwan.y@psu.ac.th; 3 Department of Pediatrics, Faculty of Medicine Ramathibodi Hospital, Mahidol University, Bangkok 10400, Thailand

**Keywords:** invasive candidiasis, pediatric, *Candida tropicalis*, antifungal susceptibility, epidemiological cutoff value, mortality, competing risks, Thailand

## Abstract

Invasive candidiasis (IC) causes substantial mortality in critically ill children, yet pediatric data from Southeast Asia remain limited. We retrospectively studied 117 children aged ≤ 18 years with proven IC at a Thai tertiary center (2009–2023). Thirty-day mortality was 25.6%, rising to 47.1% in neonates; death occurred a median of 5.5 days after diagnosis. Firth penalized logistic regression identified septic shock (aOR, 4.89; 95% CI, 1.77–14.88) and thrombocytopenia (aOR, 3.74; 95% CI, 1.17–15.35) as independent mortality predictors, with septic shock remaining significant across all analytical frameworks, including Fine–Gray competing-risks analysis. Apparent mortality associated with absence of antifungal therapy reflected reverse causation: in all six untreated children who died, *Candida* was reported only 2–7 days after death. *Candida albicans* (43.6%), *C. tropicalis* (30.8%), and *C. parapsilosis* (24.8%) predominated; *C. glabrata* was absent. Non-*albicans Candida* was already common at the outset and showed no statistically detectable increase over 15 years. Among 45 consecutive pediatric bloodstream isolates (2021–2026), amphotericin B and echinocandins largely retained activity, whereas reduced azole susceptibility was concentrated in *C. tropicalis* (47.8% fluconazole-susceptible; 26.1% posaconazole wild-type). The reduced azole susceptibility of local *C. tropicalis* isolates argues for periodic reassessment of institutional susceptibility data rather than reliance on historical or external epidemiology; in comparable settings, an echinocandin or amphotericin B is a more reliable empiric choice than an azole, pending species identification and susceptibility results.

## 1. Introduction

Invasive candidiasis (IC) is a major cause of mortality and morbidity in children [1]. Its burden is concentrated in the most critically ill, with incidence density ranging from 32.6 per 100,000 inpatient-days on general pediatric wards to 147.2 in the pediatric intensive care unit [2]. Although large reviews report a declining incidence of pediatric IC over the past 15 years [3], referral centers in low- and middle-income countries may not share this trend, and outcomes there remain poor: 30-day mortality reached 28.5% in a Thai pediatric cohort [4]. Together, these features mark IC as a persistent and underrecognized threat in resource-limited settings.

Early diagnosis of IC is challenging because culture results are delayed, making timely empiric therapy critical. The appropriate empiric choice depends on the local species distribution and resistance profile. A shift toward non-*albicans Candida* (NAC) species has been widely reported across Asia, including Thailand [4], and matters clinically because NAC species such as *C. tropicalis* are increasingly azole-resistant. However, most reports are cross-sectional or aggregate adult and pediatric data, and few test this temporal claim against longitudinal, single-population data; mortality analyses, in turn, often rely on methods ill-suited to the small number of events and may misread reverse causation as risk.

Because data on pediatric IC in Southern Thailand remain limited, we evaluated the epidemiology, incidence density, clinical characteristics, antifungal susceptibility, and predictors of 30-day mortality among neonates and children with proven IC at a tertiary teaching hospital, applying penalized regression and competing-risks methods suited to sparse events and testing the assumed temporal shift in species distribution against 15-year longitudinal data.

## 2. Materials and Methods

### 2.1. Study Design and Population

This retrospective cohort study was conducted at Songklanagarind Hospital, a tertiary referral center in Southern Thailand, between 1 January 2009 and 31 December 2023.

Case ascertainment was culture-based, not code-based, avoiding losses from ICD-10 miscoding. The institutional Digital Innovation and Data Analytics (DIDA) unit and the clinical microbiology laboratory jointly retrieved from the laboratory information system every patient aged ≤ 18 years with *Candida* isolated from a sterile site during this period, restricted to the first episode per patient. Each record was reviewed against established criteria for proven invasive candidiasis [5], requiring at least one of the following:

Candidemia: isolation of *Candida* species from at least one blood culture in a patient with clinical signs and symptoms of infection.

Deep-seated candidiasis: isolation of *Candida* from a normally sterile site (e.g., cerebrospinal, pleural, or peritoneal fluid) with a compatible infectious process.

Histologically documented candidiasis: presence of yeast cells or hyphae confirmed by histopathology from a biopsy or needle aspiration of normally sterile tissue.

Patients with *Candida* isolated only from non-sterile sites, such as respiratory or urinary specimens, without evidence of invasive infection were excluded, as were those with incomplete clinical records. The laboratory search returned 118 patients; on specimen-source review, one child whose only positive culture was middle-ear pus—not a sterile site under the consensus criteria [5], with no candidemia—was excluded, leaving 117 for analysis (Figure S2).

### 2.2. Data Collection and Outcomes

Baseline demographics, comorbidities, and clinical parameters at diagnosis were extracted from electronic medical records via a standardized REDCap form (REDCap, Vanderbilt University, Nashville, TN, USA). Gastrointestinal disease comprised bowel perforation, intestinal obstruction, midgut malrotation, short bowel syndrome, anorectal malformation, inflammatory bowel disease, or necrotizing enterocolitis. Variables included pre-diagnosis risk factors (central venous catheters, total parenteral nutrition, mechanical ventilation, and immunocompromising conditions), presentation (fever, septic shock, and thrombocytopenia), and antifungal therapy. Analyzed as a risk factor, *Candida* colonization denoted isolation of *Candida* from one or more non-sterile sites in a patient who separately met the criteria for proven IC, and is therefore distinct from the colonization-only pattern that led to exclusion. The primary outcome was 30-day all-cause mortality.

### 2.3. Antifungal Susceptibility Testing

Isolates were identified by standard biochemical tests throughout the study period; from 2017 onward, these identifications were additionally confirmed by matrix-assisted laser desorption/ionization time-of-flight mass spectrometry (MALDI-TOF MS; Bruker Daltonics GmbH, Bremen, Germany), which resolves cryptic species such as *C. orthopsilosis* and *C. pseudohaemulonii*. All 45 isolates in the contemporary susceptibility series (2021–2026) were identified by MALDI-TOF MS. Antifungal susceptibility testing (AST) used the Sensititre YeastOne (SYO) YO10 broth microdilution panel (Thermo Fisher Scientific/TREK Diagnostic Systems, Cleveland, OH, USA) covering nine agents, with standard ATCC quality-control strains. SYO is a commercial method rather than the CLSI reference assay: categorical agreement is high for azoles, but it yields higher amphotericin B minimum inhibitory concentrations (MICs) and reduced agreement for echinocandins, particularly caspofungin.

Systematic AST was not performed for most of the cohort period; between 2009 and 2023, it was requested at clinician discretion and available for only 14 of 121 cohort isolates (11.6%), too sparse to be representative. To describe the institution’s current profile, we analyzed a separate, consecutive, laboratory-complete series of all 45 *Candida* bloodstream isolates from patients aged ≤ 18 years tested between 23 December 2021 and 7 May 2026. Because some isolates came from children who also contributed to the cohort, the datasets are reported independently and never pooled, and no temporal trend is inferred.

MICs were interpreted hierarchically (detailed in Table S1). Where CLSI clinical breakpoints exist (M27M44S-Ed3; fluconazole, voriconazole, and echinocandins), isolates were classified as S, SDD, I, or R. Where none exist (amphotericin B, itraconazole, and posaconazole), CLSI M57S-Ed4 epidemiological cutoff values (ECVs) classified isolates as wild-type (WT) or non-wild-type (NWT); an ECV separates isolates without acquired resistance from those harboring it, without predicting clinical response. Agents lacking both criteria—flucytosine and all agents for *C. pseudohaemulonii*, *C. orthopsilosis*, and *C. guilliermondii*—were reported as not interpretable (NI). Because caspofungin shows substantial interlaboratory variability, non-susceptible results were corroborated against anidulafungin and micafungin. Voriconazole was unreported for five *C. tropicalis* isolates, giving a denominator of 18.

### 2.4. Statistical Analysis

Continuous variables are presented as medians with interquartile ranges (IQRs) and compared using the Mann–Whitney U test. Categorical variables are reported as frequencies and percentages and compared using the chi-square test or Fisher’s exact test when expected counts were <5. There were no missing data for modeled variables, age, sex, or platelet count. Results from selectively performed investigations were analyzed using the number examined as the denominator, without imputation. All tests were two-sided, with *p* < 0.05 considered statistically significant. Univariable analyses were exploratory, and no adjustment was made for multiple comparisons.

Primary model. Because there were only 30 deaths and one covariate showed quasi-complete separation, we used Firth’s penalized-likelihood logistic regression to reduce small-sample bias and obtain finite estimates. Adjusted odds ratios (aORs) are reported with profile penalized-likelihood 95% confidence intervals (CIs) and penalized likelihood-ratio test (PLRT) *p*-values. Wald intervals were not used because they are unreliable near separation. Four covariates were pre-specified on clinical grounds: septic shock, thrombocytopenia (platelet count <150,000/µL), mechanical ventilation, and *Candida* colonization, corresponding to 7.5 events per variable; all four also proved to have univariable *p* < 0.10. Variables assessed only in selected patients, including funduscopic findings, echocardiographic findings, and deep-seated microabscesses, were excluded from the model.

Handling of “no antifungal treatment.” This variable was excluded before modeling because untreated status was determined by death rather than by a treatment decision (Section 3.2); including it would encode reverse causation and inflate performance, and the magnitude of that inflation is quantified in a pre-specified sensitivity analysis.

Sensitivity analyses (all pre-specified) comprised: (1) unpenalized regression; (2) exclusion of post mortem diagnoses; (3) a parsimonious model (septic shock and thrombocytopenia); (4) addition of primary immunodeficiency; and (5) reintroduction of “no antifungal treatment” to quantify its inflation of estimates and discrimination. Discrimination was quantified by the area under the receiver-operating-characteristic curve (AUC) and an optimism-corrected AUC from Harrell’s bootstrap (2000 resamples).

Time-to-event analyses. With diagnosis as day 0, 30-day survival was estimated by Kaplan–Meier estimation, overall and stratified by the primary covariates, neonatal status, and *C. albicans* versus any NAC infection, compared by log-rank test. Because discharge or transfer alive before day 30 is potentially informative censoring, it was also modeled as a competing event using Aalen–Johansen cumulative incidence functions and a Fine–Gray subdistribution hazard model; as every patient had a recorded outcome date, this reduces to a Cox model with robust standard errors, with follow-up set to zero for post mortem diagnoses.

Temporal trends and incidence density. Species proportions and 30-day mortality were compared between early (2009–2016) and late (2017–2023) periods by Fisher’s exact test and across three five-year periods by the Cochran–Armitage trend test. Incidence density (proven IC cases per 100,000 pediatric inpatient-days) was reported with exact Poisson CIs and its trend assessed by Poisson regression, with a negative-binomial model checking overdispersion. Denominators (total pediatric ward inpatient-days) existed only from 2017, so this analysis covers 2017–2023 (60 of 117 cases).

Software and generative AI use. Analyses were performed in Python 3.12 (Python Software Foundation, Wilmington, DE, USA), with the Firth implementation validated against maximum-likelihood and closed-form penalized estimates. Claude (Anthropic, San Francisco, CA, USA, 2026) assisted with English-language editing and figure preparation, while ChatGPT (GPT-5.6 Thinking, OpenAI, 2026; accessed 16 July 2026) assisted with the design and generation of the graphical abstract. Neither tool was used for study design, data collection, statistical analysis, or interpretation. All AI-assisted outputs were reviewed and verified by the authors, who take full responsibility for the final content.

## 3. Results

### 3.1. Patient Characteristics and Clinical Presentation

A total of 117 pediatric patients with proven invasive candidiasis were included (Figure S2). Baseline demographic, clinical, and treatment characteristics, stratified by 30-day survival status, are presented in Table 1.

The median age was 2.3 years (IQR, 0.2–7.7) and 17 patients (14.5%) were neonates; 53.8% were male. Underlying disease was present in 112 (95.7%), most commonly congenital heart disease, hematologic malignancy, gastrointestinal disease, and prematurity (Table 1). At presentation, 81.2% had fever, 43.6% septic shock, and 67.5% thrombocytopenia (median platelet count 89.0 × 10^3^/µL). Screening for dissemination was selective, so findings are reported per examined subgroup: deep-seated microabscesses in 15 of 52 children who underwent abdominal imaging (28.8%), chorioretinitis or endophthalmitis in 2 of 38 examined by funduscopy (5.3%), and endocarditis in 1 of 33 examined by echocardiography (3.0%).

Major pre-diagnosis risk factors included central venous catheter (58.1%), *Candida* colonization (59.8%), and mechanical ventilation (53.0%) (Table 1). Prior antifungal exposure was recorded only for the 105 children who received antifungal treatment; of these, 6 (5.7%) had been exposed before diagnosis, all with non-*albicans* infection (three *C. tropicalis*, three *C. parapsilosis*).

Blood was the most common source (110/117; 94.0%); other sterile sites were infrequent (peritoneal 13.7%, other tissue 7.7%, pleural 3.4%, CSF 1.7%, vitreous 0.9%), and 21 children had multiple positive sites (Table S2). Among 105 treated children, amphotericin B and fluconazole were the most frequently used agents, and most received more than one agent sequentially (Table S3).

### 3.2. Untreated Patients and the Timing of Culture Reporting

Of the 117 children, 105 (89.7%) received antifungal treatment and 12 (10.3%) did not—six of the untreated died and six survived. In every fatal untreated case, the *Candida* culture was reported only 2–7 days after death (median 4), so all six were post mortem diagnoses reflecting slow growth on solid medium; absence of therapy was thus a consequence, not a cause, of death. Among survivors, two had transient candidemia not requiring treatment and four had cultures reported on or after discharge. Thirty-day mortality among treated patients was 22.9% (24/105).

### 3.3. Timing and Pattern of Death

**Figure 1:**
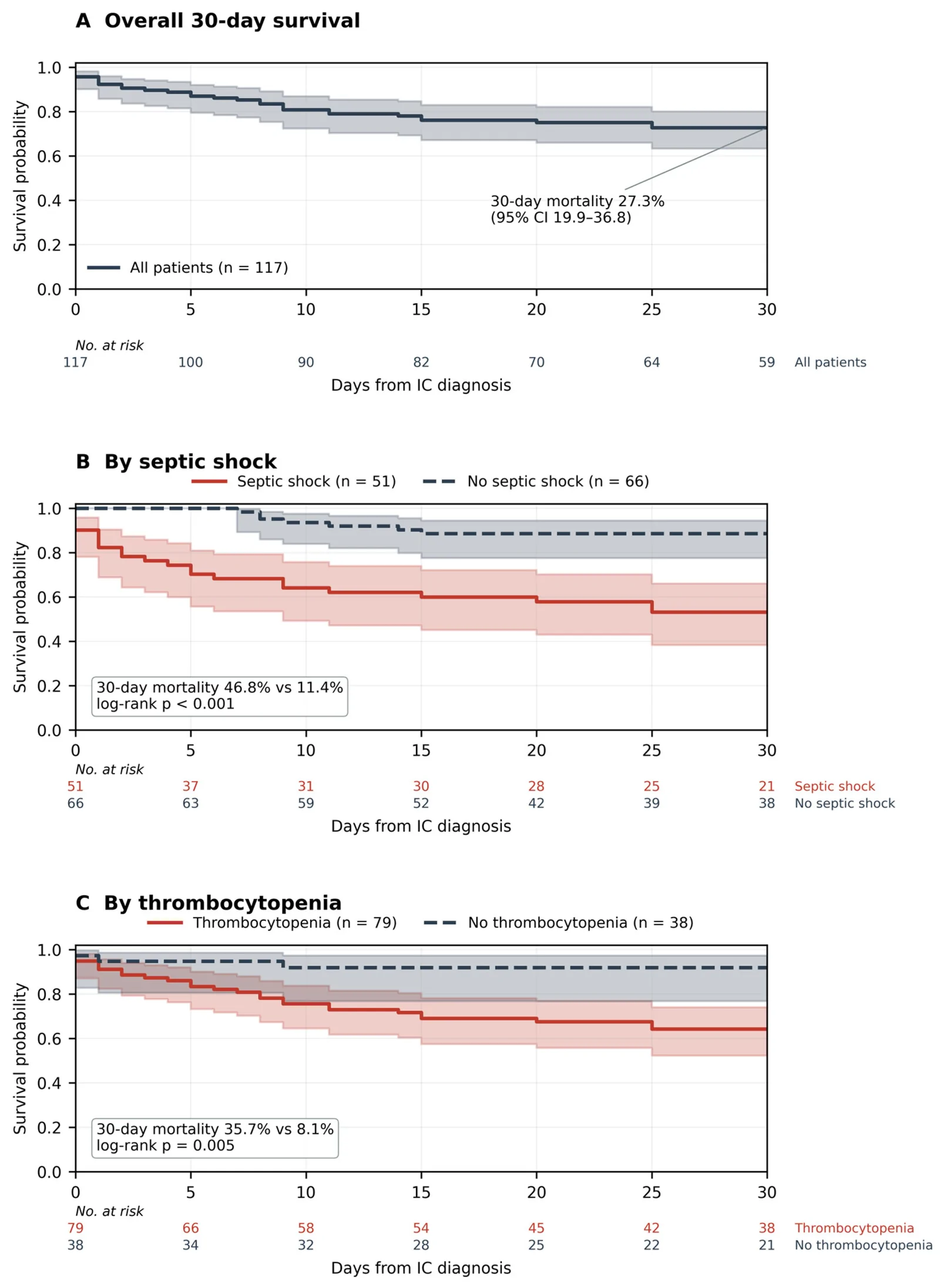
Kaplan–Meier 30-day survival of pediatric invasive candidiasis (*N* = 117), measured from the date of diagnosis. (**A**) Overall survival; the 30-day mortality estimate was 27.3% (95% CI, 19.9–36.8%). (**B**) Survival stratified by septic shock. (**C**) Survival stratified by thrombocytopenia. Shaded areas are 95% confidence intervals; the number of patients at risk is shown beneath each panel, and within-panel labels give the Kaplan–Meier 30-day mortality in each stratum with the log-rank *p*-value.

Thirty of 117 children (25.6%) died within 30 days, with no loss to follow-up; the Kaplan–Meier estimate was 27.3% (95% CI, 19.9–36.8%) (Figure 1A), and mortality reached 47.1% (8/17) in neonates. Death occurred a median of 5.5 days after diagnosis (IQR, 1–10.5), 37% within 48 h and 57% within 7 days. Because 28 children were discharged alive before day 30, the naive Kaplan–Meier estimator modestly overstates mortality; the competing-risks (Aalen–Johansen) cumulative incidence of death at day 30 was 24.8% (95% CI, 17.1–33.3%).

Thirty-day mortality differed by septic shock (Kaplan–Meier estimates, 46.8% vs. 11.4%; log-rank *p* < 0.001), thrombocytopenia (35.7% vs. 8.1%; *p* = 0.005), mechanical ventilation (35.7% vs. 17.2%; *p* = 0.027), and neonatal status (53.0% vs. 22.7%; *p* = 0.027) (Figure 1B,C). Differences by *Candida* colonization were borderline (34.5% vs. 15.2%; *p* = 0.061), and mortality did not differ between any NAC infection and *C. albicans* alone (25.7% vs. 29.6%; *p* = 0.647).

### 3.4. Independent Predictors of 30-Day Mortality

In the primary Firth model (four pre-specified covariates; 7.5 events per variable), septic shock (aOR 4.89; 95% CI, 1.77–14.88) and thrombocytopenia (aOR 3.74; 1.17–15.35) were independently associated with mortality; mechanical ventilation and *Candida* colonization were not (Table 2). Both predictors stayed significant in all five pre-specified sensitivity analyses, including unpenalized estimation, exclusion of the six post mortem diagnoses, a parsimonious two-covariate model, and the addition of primary immunodeficiency, which was present in eight children, none of whom died; Firth penalization accommodates the resulting quasi-complete separation (Table 3).

Reintroducing “no antifungal treatment” illustrates the reverse-causation artifact: the adjusted OR for thrombocytopenia doubled, *Candida* colonization crossed significance, and apparent discrimination rose from 0.81 to 0.83 (Table 3 and model S5). In the competing-risks framework, the Fine–Gray model reproduced the finding for septic shock (subdistribution hazard ratio [sHR] 4.27; 95% CI, 1.60–11.40), whereas thrombocytopenia was of similar magnitude but less precise (sHR 3.22; 0.84–12.31). Septic shock was therefore the only predictor significant under every analytical framework.

### 3.5. Incidence Density

Over 2017–2023, 60 IC cases occurred during 170,721 pediatric inpatient-days, a pooled incidence density of 35.1 per 100,000 inpatient-days (95% CI, 26.8–45.2). Annual estimates ranged from 10.8 in 2019 (three cases) to 48.4 in 2021 (nine cases) (Figure S1). Poisson regression showed no temporal trend (incidence rate ratio 1.009 per year; 95% CI, 0.892–1.141; *p* = 0.891), with a negative-binomial model giving a materially identical estimate. The low case count in 2019 (*n* = 3) yields a wide interval and drives most year-to-year variation.

### 3.6. Candida Species Distribution and Temporal Trends

The 117 patients yielded 121 isolates (four patients, 3.4%, had mixed cultures). By patient, *C. albicans* was identified in 51 (43.6%), *C. tropicalis* in 36 (30.8%), *C. parapsilosis* in 29 (24.8%), *C. krusei* in 3 (2.6%), and *C. guilliermondii* in 2 (1.7%); non-*albicans* species accounted for 57.9% of the 121 isolates. *C. glabrata*, *C. lusitaniae*, *C. auris*, and *C. dubliniensis* were not identified.

Non-*albicans* species predominated throughout the 15-year span. The NAC proportion was 59.6% (2009–2016) versus 58.3% (2017–2023) (*p* = 1.000), with no monotonic trend (Cochran–Armitage *p* = 0.878); *C. tropicalis*, *C. parapsilosis*, and *C. albicans* showed no significant shift (all *p* > 0.39), and 30-day mortality was unchanged (28.1% vs. 23.3%; *p* = 0.673). NAC species thus posed a persistently high burden, without a statistically detectable increase over the study period.

### 3.7. Antifungal Susceptibility

Between December 2021 and May 2026, 45 consecutive pediatric *Candida* bloodstream isolates underwent susceptibility testing: *C. tropicalis* (*n* = 23; 51.1%), *C. parapsilosis* (*n* = 10; 22.2%), *C. albicans* (*n* = 7; 15.6%), *C. pseudohaemulonii* (*n* = 3), *C. orthopsilosis* (*n* = 1), and *C. guilliermondii* (*n* = 1). The last three lack CLSI interpretive criteria for all nine agents and are reported as not interpretable, although their MIC distributions are tabulated (Table S1).

Amphotericin B was wild-type against every interpretable isolate. All *C. albicans* and *C. tropicalis* isolates were susceptible to anidulafungin and micafungin, and caspofungin susceptibility was 87.0% (20/23) in *C. tropicalis*. In *C. parapsilosis*, anidulafungin and micafungin susceptibility were each 90% (9/10), consistent with the intrinsically elevated echinocandin MICs of this species rather than acquired resistance.

**Figure 2:**
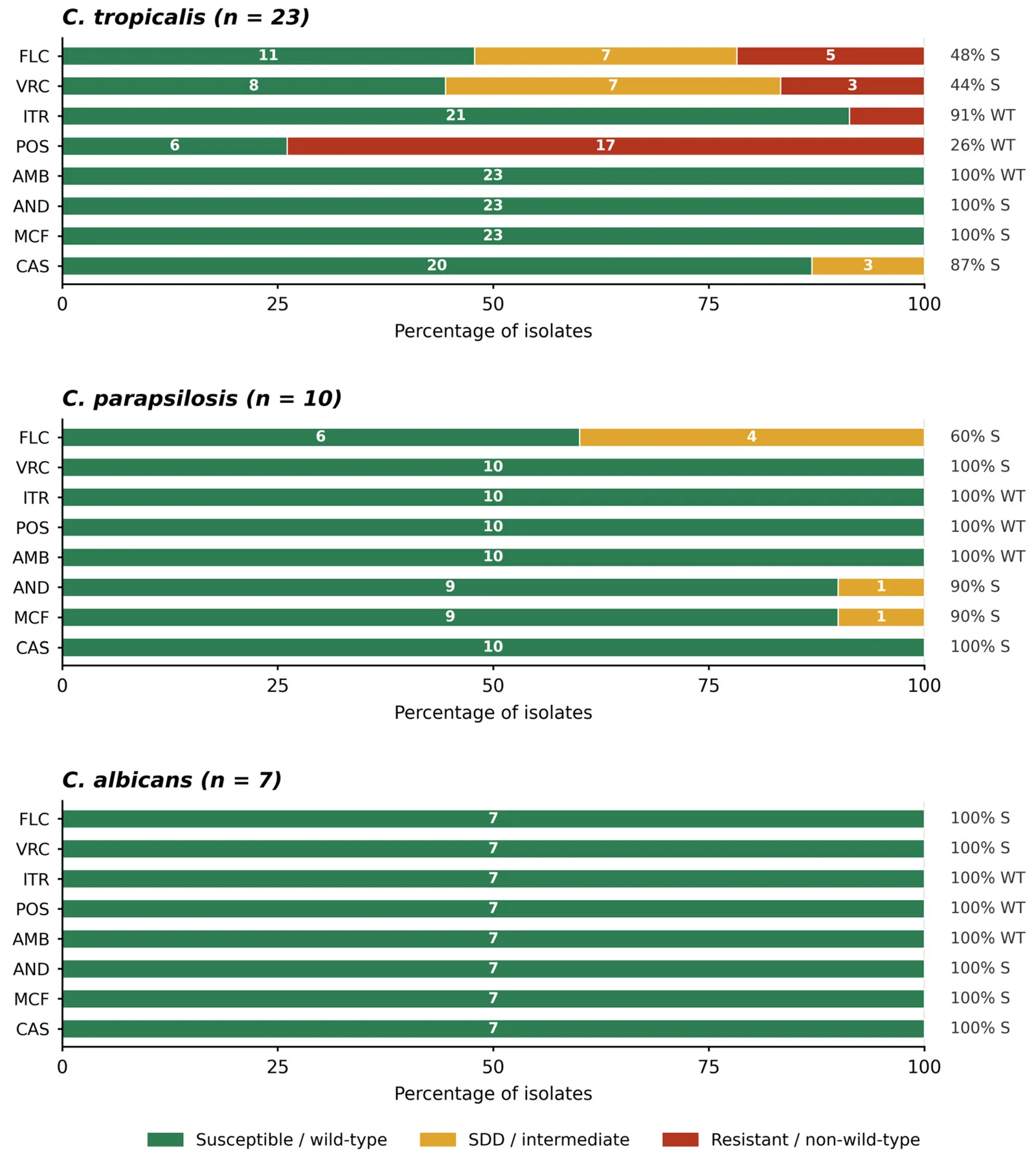
Antifungal susceptibility of 40 of 45 pediatric *Candida* bloodstream isolates with CLSI interpretive criteria, by species, December 2021–May 2026. Bars give the percentage of isolates in each interpretive category, with the isolate count printed on each segment and the favorable percentage (susceptible or wild-type) at the right margin; the voriconazole row for *C. tropicalis* has a denominator of 18 (see Section 2.3). Categories were assigned as described in Section 2.3. The remaining five isolates (three *C. pseudohaemulonii*, one *C. orthopsilosis*, and one *C. guilliermondii*) lack interpretive criteria for all agents and are not shown; their MIC distributions are given in Table S1. FLC, fluconazole; VRC, voriconazole; ITR, itraconazole; POS, posaconazole; AMB, amphotericin B; AND, anidulafungin; MCF, micafungin; CAS, caspofungin.

Reduced azole susceptibility was concentrated in *C. tropicalis*: 47.8% (11/23) were fluconazole-susceptible (MIC_50_ 4, MIC_90_ 64 µg/mL), 44.4% (8/18) voriconazole-susceptible, and 26.1% (6/23) posaconazole wild-type, whereas itraconazole wild-type remained high (91.3%). Because voriconazole was unreported for five isolates, four of them fluconazole non-susceptible, the susceptible proportion across all 23 is bounded between 34.8% and 56.5% and may overstate activity. By contrast, all seven *C. albicans* isolates were susceptible or wild-type to every agent, and *C. parapsilosis* was fully voriconazole-susceptible though only 60% fluconazole-susceptible (Figure 2; Table S1).

## 4. Discussion

This 15-year pediatric cohort highlights three key findings: persistent non-*albicans Candida* predominance without a statistically detectable temporal increase, reverse causation underlying the apparent association between no antifungal therapy and mortality, and reduced azole susceptibility concentrated in *C. tropicalis* despite largely preserved amphotericin B and echinocandin activity. Together, these findings link long-term local epidemiology with contemporary susceptibility data to inform empiric antifungal therapy in a setting with limited pediatric evidence.

Our 30-day mortality of 25.6% was comparable to the 28.5% reported from King Chulalongkorn Memorial Hospital in Bangkok in an earlier era [4] and higher than the 16.6% subsequently reported from the same center [6], as well as the 19.7% of a Jerusalem cohort [7]. A large contemporary Turkish pediatric cohort reported a lower mortality of 10.8% [8]. Differences in age eligibility and case mix likely contribute to these variations. Compared with the more recent Bangkok cohort [6], our patients had more septic shock (43.6% vs. 18.0%) and prematurity (18.8% vs. 10.9%), while the Turkish cohort excluded neonates, who had the highest mortality in our study (47.1%). The high mortality in our cohort underscores the importance of timely diagnosis and severity of illness in addition to appropriate antifungal therapy.

IC occurred across a broad range of underlying conditions, including hematologic malignancy, congenital heart disease, gastrointestinal disease, prematurity, and central venous or peritoneal catheter exposure. A similar pattern was reported in the Bangkok pediatric cohort [6] and is consistent with established pediatric IC risk factors, including gastrointestinal barrier disruption, vascular access, and parenteral nutrition [2].

Firth penalized regression identified septic shock (aOR, 4.89) and thrombocytopenia (aOR, 3.74) as independent mortality predictors, consistent with a previous Thai pediatric cohort [4]. Septic shock remained significant in all sensitivity and Fine–Gray analyses (sHR, 4.27), making it the most robust predictor. Thrombocytopenia showed a similar but less precise association in the Fine–Gray model (sHR, 3.22). Because both are evident before culture confirmation, they may help identify children requiring intensive monitoring and heightened consideration of empiric antifungal therapy in the appropriate clinical context. Thrombocytopenia may reflect severe inflammation, platelet consumption, coagulopathy, or impaired bone-marrow reserve and also predicted mortality in adult candidemia [9,10].

That same Thai pediatric cohort [4] also identified no antifungal treatment as an independent mortality predictor, but our data indicate reverse causation. In all six untreated children who died, the *Candida* culture was reported 2–7 days after death (median 4), so diagnosis occurred too late for treatment rather than treatment being withheld. Reintroducing this variable inflates its own aOR to 7.17, doubles that for thrombocytopenia (3.74 → 7.55), and raises the apparent AUC (0.81 → 0.83) without representing a modifiable exposure. This diagnostic delay underscores the need for earlier recognition and rapid culture-independent diagnostics, such as T2*Candida* and *Candida* PCR, which can shorten the time to *Candida* detection in pediatric patients [11,12].

Species distribution differed substantially among pediatric centers. Although *C. albicans* remained the most common individual species in our cohort (43.6%), NAC collectively predominated, mainly *C. tropicalis* (30.8%) and *C. parapsilosis* (24.8%). *C. tropicalis* accounted for 29.5% of infections in the recent Bangkok cohort [6], compared with 12.9% in Jerusalem [7] and 5.8% in the larger Turkish series [8]. In contrast, *C. parapsilosis* accounted for 46.0% of isolates in the Turkish cohort [8]. *C. glabrata* was absent from our cohort but accounted for 4.9% and 8.4% of isolates in the Bangkok and Jerusalem cohorts, respectively [6,7]; its absence here is consistent with lower regional azole selection pressure [13]. These differences likely reflect variation in age, underlying conditions, antifungal exposure, device use, referral patterns, and local fungal epidemiology [14,15,16,17] and illustrate why species distributions from one institution cannot be assumed to represent another.

Species-level identification is clinically relevant because conventional phenotypic and biochemical methods may misidentify closely related *Candida* species complexes. Compared with rDNA sequencing as a reference method, MALDI-TOF MS provides rapid, high-throughput identification and improves resolution of species within complexes such as the *C. parapsilosis* complex [18,19]. The two approaches differ mainly in speed, cost, and scope. MALDI-TOF requires under ten minutes of sample preparation and spectral acquisition, is largely automated, and costs little per isolate, but can identify only organisms represented in its reference database [19,20]. Sequencing of the ITS region or the D1/D2 domain of the LSU rRNA gene resolves cryptic and newly described species without that constraint and serves as the reference standard but is more expensive, more labor-intensive, and slower [20]. In practice, low-confidence or discordant MALDI-TOF results are therefore referred for sequencing [18,19,20]. In our study, MALDI-TOF confirmation from 2017 onward, including all 45 isolates in the contemporary susceptibility series, strengthens confidence in species identification. However, pre-2017 isolates were identified by biochemical methods alone; therefore, cryptic species may have been under-recognized and residual species-level misclassification cannot be excluded.

Among the 45 contemporary isolates, amphotericin B retained wild-type activity against all interpretable isolates, and all tested *C. albicans* and *C. tropicalis* isolates were susceptible to micafungin and anidulafungin. The 90% echinocandin susceptibility observed in *C. parapsilosis* should be interpreted cautiously because of the intrinsically higher echinocandin MIC distribution of this species and the small number of isolates. The principal susceptibility concern was *C. tropicalis*: only 47.8% of isolates were fluconazole-susceptible, 44.4% were voriconazole-susceptible, and 26.1% were posaconazole wild-type. The posaconazole findings require particular caution because 10 of 17 non-wild-type isolates had MICs only one dilution above the ECV (0.25 vs. 0.12 µg/mL), which may partly reflect variability around the epidemiological cutoff rather than clinical resistance.

The marked reduction in azole susceptibility among *C. tropicalis* isolates is consistent with growing regional concern regarding azole-resistant *C. tropicalis* [21] and appears to involve multiple molecular pathways rather than a single resistance mechanism. Reported mechanisms include *ERG11* mutations or overexpression, increased expression of efflux transporters such as *CDR1*, *CDR3*, and *MDR1*, altered transcriptional regulation involving *TAC1* and *UPC2*, and broader changes in ergosterol-biosynthesis pathways [22]. In one molecular study, only 12 of 32 azole-resistant *C. tropicalis* isolates carried nonsynonymous *ERG11* mutations, whereas resistant isolates without these mutations showed greater expression of efflux- and ergosterol-pathway genes [22]. A Thai bloodstream-isolate study identified *ERG11* missense mutations in 83% of fluconazole-resistant *C. tropicalis* isolates; notably, resistant isolates without *ERG11* mutations showed higher *CDR1* expression, suggesting that alternative mechanisms may contribute to resistance [23]. Similar mechanisms, including *ERG11* target alterations and overexpression and increased activity of Cdr1p/Cdr2p and Mdr1p efflux pumps, are well described in *C. albicans* [24,25]. Therefore, the lower azole susceptibility observed in *C. tropicalis* in our contemporary series should not be attributed to a single species-specific pathway. Because molecular resistance testing was not performed in our isolates, these published mechanisms remain plausible explanations rather than mechanisms demonstrated directly in our cohort.

The species difference in azole susceptibility is also supported by other Thai pediatric data. A recent cohort from King Chulalongkorn Memorial Hospital reported greater azole resistance among *C. tropicalis* than *C. albicans*, although the interpretive criteria differed from those used in our study, and direct comparison of percentages is therefore inappropriate [6]. An earlier report from the same Bangkok center reported susceptibility for only 39 of 102 episodes and grouped non-*albicans* species together, so *C. tropicalis* cannot be separated from it [4]. Reduced fluconazole susceptibility among *C. tropicalis* has also been documented in an adult-inclusive series from this institution over an overlapping period [26].

These findings have direct implications for empiric treatment at our center. With fewer than half of contemporary *C. tropicalis* isolates susceptible to fluconazole, empiric azole monotherapy cannot be reliably expected to cover this species in critically ill children with suspected IC. IDSA guidance considers fluconazole an initial option primarily for selected non-critically ill patients unlikely to harbor a fluconazole-resistant species, reserving it otherwise for step-down therapy once the patient is stable and the isolate susceptible [27]; our local profile therefore supports an echinocandin or amphotericin B while species identification and susceptibility results are pending. In neonates, the highest-mortality group in this cohort, amphotericin B deoxycholate remains an appropriate first-line option, particularly before central nervous system involvement has been excluded [27,28].

This variation in species distribution and susceptibility among centers also supports local cumulative surveillance rather than reliance on external antibiograms. CLSI M39 recommends that routine cumulative susceptibility reports be analyzed and presented at least annually and generally include ≥30 isolates per species; for yeast species with fewer than 30 isolates, data from more than one year may be combined [29,30]. In low-volume pediatric populations such as ours, annual review of newly accumulated isolates together with a rolling multi-year species-specific summary may therefore be more informative than stand-alone annual percentages based on small denominators. Empiric treatment protocols should be reconsidered when accumulated local data indicate a clinically meaningful change in species distribution or antifungal susceptibility, or earlier if a new resistant phenotype or epidemiologic cluster emerges.

Beyond the primary findings, two secondary observations warrant consideration. First, endophthalmitis was found in only 2 of 38 children undergoing funduscopy (5.3%), consistent with the ~3% reported in pediatric candidemia [31,32]. This low yield underlies the divergence between the 2016 IDSA recommendation for routine dilated retinal examination and the American Academy of Ophthalmology recommendation against routine screening in asymptomatic patients [33]; because critically ill and very young children may be unable to report visual symptoms reliably, an individualized approach may be appropriate.

Second, mortality was lower among children with *Candida* microabscesses (6.7%, 1/15) than among imaged children without microabscesses (18.9%, 7/37) or those not imaged (33.8%). This finding should not be interpreted as a protective association because abdominal imaging was performed in only 52 of 117 children. Selection, survivorship, and immortal-time biases are more plausible explanations; hepatosplenic candidiasis is often detected during or after neutrophil recovery, and patients must survive long enough for imaging and diagnosis [34,35,36].

Several limitations should be considered. This retrospective single-center study is susceptible to referral bias and may not be generalizable beyond tertiary-care settings. The modest number of mortality events limited multivariable modeling. We therefore restricted the primary model to four clinically prespecified covariates and used Firth penalization to reduce small-sample bias and address quasi-complete separation; the results should be interpreted as cohort-specific mortality associations rather than as a validated prediction model. Antifungal susceptibility data were derived from a separate consecutive series collected from December 2021 to May 2026, used Sensititre YeastOne rather than reference broth microdilution, and lacked voriconazole results for several isolates; these data therefore describe the contemporary local susceptibility profile rather than a temporal resistance trend. MALDI-TOF confirmation was available only from 2017 onward, so residual species-level misclassification among earlier isolates identified biochemically cannot be excluded.

In addition, findings from selectively investigated subgroups, including funduscopy, echocardiography, and abdominal imaging, are susceptible to selection and survivorship bias. The study was also underpowered to detect modest temporal changes in the NAC proportion: the early-to-late difference was −1.3 percentage points, but the wide 95% CI (−19.1 to +16.5) means that a clinically meaningful increase cannot be excluded. A larger 18-year pediatric cohort detected a temporal increase in NAC species (odds ratio 1.079 per year; 95% CI, 1.032–1.128) [8], which our smaller cohort may not have identified. Finally, discharge or transfer alive before day 30 could have introduced informative censoring, although this was addressed using competing-risks analysis.

## 5. Conclusions

In this 15-year pediatric cohort, invasive candidiasis was associated with a substantial 30-day mortality of 25.6%. Septic shock and thrombocytopenia at presentation identified children at highest risk, and septic shock remained significant under every analytical framework. Reduced azole susceptibility was concentrated in *C. tropicalis*, whereas amphotericin B and echinocandins generally retained activity. We propose three concrete steps for centers caring for critically ill children: identify every sterile-site *Candida* isolate to species level; perform susceptibility testing on bloodstream isolates where capacity permits; and compile both into a pediatric-specific antibiogram, reviewed at least annually and, where annual isolate numbers fall below the threshold recommended for cumulative reporting, pooled across more than one year. In critically ill children, an echinocandin or amphotericin B may be preferred until susceptibility results are available, while amphotericin B deoxycholate remains an appropriate first-line option for neonatal candidiasis.

## Figures and Tables

**Table 1:** Baseline characteristics of 117 children with proven invasive candidiasis, by 30-day survival status.

Characteristic	All Patients (*n* = 117)	Survivors (*n* = 87)	Non-Survivors (*n* = 30)	*p*-Value
**Demographics**				
Age (years), median (IQR)	2.3 (0.2–7.7)	1.5 (0.2–6.3)	4.1 (0.1–11.0)	0.264
Neonate (<1 month), *n* (%)	17 (14.5)	9 (10.3)	8 (26.7)	0.029
Male, *n* (%)	63 (53.8)	51 (58.6)	12 (40.0)	0.078
Body weight (kg), median (IQR)	9.3 (3.3–22.5)	7.7 (3.5–18.4)	14.8 (2.7–29.8)	0.330
**Underlying conditions**				
Any underlying disease, *n* (%)	112 (95.7)	83 (95.4)	29 (96.7)	1.000
Hematologic malignancy, *n* (%)	25 (21.4)	15 (17.2)	10 (33.3)	0.064
Solid tumor, *n* (%)	12 (10.3)	8 (9.2)	4 (13.3)	0.501
Primary immunodeficiency, *n* (%)	8 (6.8)	8 (9.2)	0 (0.0)	0.111
Secondary immunodeficiency, *n* (%)	9 (7.7)	6 (6.9)	3 (10.0)	0.692
Congenital heart disease, *n* (%)	26 (22.2)	17 (19.5)	9 (30.0)	0.235
Chronic lung disease, *n* (%)	21 (17.9)	18 (20.7)	3 (10.0)	0.272
Neurologic disease, *n* (%)	12 (10.3)	9 (10.3)	3 (10.0)	1.000
Gastrointestinal disease, *n* (%)	25 (21.4)	21 (24.1)	4 (13.3)	0.303
Prematurity (GA < 37 weeks), *n* (%)	22 (18.8)	15 (17.2)	7 (23.3)	0.462
Malnutrition, *n* (%)	7 (6.0)	6 (6.9)	1 (3.3)	0.676
**Host- and treatment-related risk factors**				
Neutropenia (ANC < 1000/µL), *n* (%)	34 (29.1)	22 (25.3)	12 (40.0)	0.126
Immunosuppressive therapy, *n* (%)	35 (29.9)	22 (25.3)	13 (43.3)	0.063
Any catheter, *n* (%)	81 (69.2)	60 (69.0)	21 (70.0)	0.916
Central venous catheter, *n* (%)	68 (58.1)	49 (56.3)	19 (63.3)	0.502
Peritoneal drain, *n* (%)	19 (16.2)	13 (14.9)	6 (20.0)	0.517
Mechanical ventilation, *n* (%)	62 (53.0)	41 (47.1)	21 (70.0)	0.030
Total parenteral nutrition, *n* (%)	44 (37.6)	34 (39.1)	10 (33.3)	0.575
Surgery on ICU admission, *n* (%)	34 (29.1)	24 (27.6)	10 (33.3)	0.550
*Candida* colonization, *n* (%)	70 (59.8)	47 (54.0)	23 (76.7)	0.029
**Clinical presentation and laboratory findings**				
Fever, *n* (%)	95 (81.2)	70 (80.5)	25 (83.3)	0.728
Septic shock, *n* (%)	51 (43.6)	28 (32.2)	23 (76.7)	<0.001
Thrombocytopenia (<150,000/µL), *n* (%)	79 (67.5)	52 (59.8)	27 (90.0)	0.003
Platelet count (×10^3^/µL), median (IQR)	89.0 (38.0–190.0)	109.0 (47.0–236.5)	64.5 (20.8–104.5)	0.010
Deep-seated microabscess, n/N (%)	15/52 (28.8)	14/44 (31.8)	1/8 (12.5)	0.412
***Candida* species**				
*C. albicans*, *n* (%)	51 (43.6)	37 (42.5)	14 (46.7)	0.693
*C. tropicalis*, *n* (%)	36 (30.8)	24 (27.6)	12 (40.0)	0.204
*C. parapsilosis*, *n* (%)	29 (24.8)	26 (29.9)	3 (10.0)	0.030
*C. krusei*, *n* (%)	3 (2.6)	1 (1.1)	2 (6.7)	0.161
*C. guilliermondii*, *n* (%)	2 (1.7)	2 (2.3)	0 (0.0)	1.000
Any non-*albicans Candida*, *n* (%)	69 (59.0)	52 (59.8)	17 (56.7)	0.766
**Treatment and outcome**				
Received antifungal therapy, *n* (%)	105 (89.7)	81 (93.1)	24 (80.0)	0.041
Length of hospital stay (days), median (IQR)	46.0 (21.0–75.0)	57.0 (36.0–86.5)	20.0 (7.0–30.8)	<0.001

IQR, interquartile range; *n*, number of patients; ANC, absolute neutrophil count; GA, gestational age; ICU, intensive care unit. Non-survivors are patients who died within 30 days of the diagnosis of invasive candidiasis. Percentages for categorical variables are column percentages of the group denominator shown in the header, except for deep-seated microabscess, which is reported as n/N (%) among the 52 patients who underwent abdominal imaging, since the variable was recorded only for those patients. Species categories are not mutually exclusive because four patients had two *Candida* species isolated; proportions are of patients, not isolates. Shorter stay among non-survivors reflects death rather than recovery and is not protective. Statistical tests are described in Section 2.4.

**Table 2:** Independent predictors of 30-day mortality: Firth penalized logistic regression (*N* = 117; 30 events).

Risk Factor	Crude OR (95% PL CI)	Adjusted OR (95% PL CI)	PLRT χ^2^	*p*
Septic shock	6.54 (2.67–17.67)	4.89 (1.77–14.88)	9.56	0.002
Thrombocytopenia (<150,000/µL)	5.31 (1.80–20.88)	3.74 (1.17–15.35)	5.05	0.025
Mechanical ventilation	2.54 (1.09–6.29)	1.39 (0.48–4.02)	0.37	0.541
*Candida* colonization	2.67 (1.10–7.12)	2.46 (0.92–7.18)	3.20	0.074

OR, odds ratio; PL CI, profile penalized-likelihood confidence interval; PLRT, penalized likelihood-ratio test. All estimates are from Firth penalized-likelihood logistic regression. Overall model PLRT χ^2^ = 33.50, df = 4, *p* < 0.001; apparent AUC 0.81, optimism-corrected 0.78. “No antifungal treatment” was excluded before modeling because of reverse causation (see Section 2.4 and Table 3).

**Table 3:** Pre-specified sensitivity analyses for 30-day mortality.

Model	*n* (Events)	Septic Shock, aOR (95% CI)	Thrombocytopenia, aOR (95% CI)	Apparent AUC
Primary (Firth, 4 covariates)	117 (30)	4.89 (1.77–14.88)	3.74 (1.17–15.35)	0.81
S1 Unpenalized maximum likelihood	117 (30)	5.42 (1.88–17.32)	4.35 (1.27–20.25)	0.81
S2 Excluding post mortem diagnoses	111 (24)	3.68 (1.25–11.81)	6.52 (1.48–61.33)	0.81
S3 Parsimonious (2 covariates)	117 (30)	5.65 (2.25–15.54)	4.27 (1.37–17.41)	0.77
S4 + primary immunodeficiency	117 (30)	4.77 (1.70–14.85)	4.06 (1.27–16.73)	0.83
S5 + “no antifungal treatment”	117 (30)	4.54 (1.60–14.16)	7.55 (1.81–54.27)	0.83
Fine–Gray subdistribution hazard model	117 (30)	sHR 4.27 (1.60–11.40)	sHR 3.22 (0.84–12.31)	—

Unless the row label states otherwise, all models are Firth penalized logistic regressions adjusted for septic shock, thrombocytopenia, mechanical ventilation, and *Candida* colonization; 95% CIs are profile penalized-likelihood intervals. In model S5, “no antifungal treatment” itself had an aOR of 7.17 (1.45–47.88; *p* = 0.015), and *Candida* colonization became nominally significant (aOR 3.01; *p* = 0.038), illustrating the inflation induced by this reverse-causal variable. sHR, subdistribution hazard ratio, with discharge or transfer alive before day 30 as the competing event; robust sandwich standard errors. Because censoring was purely administrative at day 30, inverse-probability-of-censoring weights equal one throughout follow-up, and the subdistribution model reduces exactly to a Cox model retaining competing-event patients in the risk set.

## Data Availability

The datasets generated and analyzed during the current study are not publicly available due to institutional and ethical restrictions on sharing individual-level pediatric patient data but are available from the corresponding author on reasonable request, subject to approval by the Human Research Ethics Committee of Prince of Songkla University.

## Supplementary Materials

The following supporting information can be downloaded at: https://www.mdpi.com/article/10.3390/jof12090664/s1. Table S1: Antifungal susceptibility of 45 consecutive pediatric *Candida* bloodstream isolates by species and agent, with CLSI interpretive categories, MIC ranges, MIC_50_ and MIC_90_. Table S2: Sites of *Candida* isolation among the 117 patients. Table S3: Antifungal agents administered to the 105 treated patients. Figure S1: Annual incidence density of pediatric invasive candidiasis, 2017–2023. Figure S2: Flow of participants through the study. File S1: STROBE checklist for cohort studies.

## Abbreviations

The following abbreviations are used in this manuscript:

5FC	Flucytosine
AMB	Amphotericin B
AND	Anidulafungin
aOR	Adjusted odds ratio
AST	Antifungal susceptibility testing
AUC	Area under the receiver-operating-characteristic curve
CAS	Caspofungin
CI	Confidence interval
CIF	Cumulative incidence function
CLSI	Clinical and Laboratory Standards Institute
ECV	Epidemiological cutoff value
FLC	Fluconazole
IC	Invasive candidiasis
ICU	Intensive care unit
IDSA	Infectious Diseases Society of America
IQR	Interquartile range
IRR	Incidence rate ratio
ITR	Itraconazole
MCF	Micafungin
MIC	Minimum inhibitory concentration
NAC	Non-*albicans Candida*
NWT	Non-wild-type
OR	Odds ratio
PL CI	Profile penalized-likelihood confidence interval
PLRT	Penalized likelihood-ratio test
POS	Posaconazole
sHR	Subdistribution hazard ratio
SDD	Susceptible-dose-dependent
SYO	Sensititre YeastOne
VRC	Voriconazole
WT	Wild-type
