# Supplementary material for: Persistent Non-albicans Predominance, High Mortality, and Azole-Non-Susceptible Candida tropicalis: A 15-Year Pediatric Invasive Candidiasis Cohort in Southern Thailand
Source: J Fungi (Basel). 2026 Sep 3;12(9):664. doi: 10.3390/jof12090664 (PMC13608856; doi:10.3390/jof12090664)
Supplement: Supplementary file 1 [file jof-12-00664-s001.zip › jof-4473530-supplementary.pdf]

Supplementary Materials

Persistent Non-*albicans* Predominance, High Mortality, and Azole-Non-Susceptible *Candida tropicalis*: A 15-Year Pediatric Invasive Candidiasis Cohort in Southern Thailand

Puttichart Khantee, Kochakorn Pinichkijpaisal, Mingkwan Yingkajorn, Therdpong Thongseiratch, Kamolwish Laoprasopwattana

Contents

Table S1. Antifungal susceptibility of 45 consecutive pediatric Candida bloodstream isolates

Table S2. Sites of Candida isolation among 117 children with proven invasive candidiasis

Table S3. Antifungal agents administered to 105 treated children

Figure S1. Annual incidence density of pediatric invasive candidiasis, 2017–2023

Figure S2. Flow of participants through the study

File S1. STROBE checklist for cohort studies

Table S1. Antifungal susceptibility of 45 consecutive pediatric *Candida* bloodstream isolates, December 2021 – May 2026

Antifungal agent	Basis	n tested	MIC range (µg/mL)	MIC50	MIC90	CLSI category (n)	S or WT, n (%)
***Candida tropicalis* (n = 23)**							
Fluconazole	BP	23	1–256	4	64	S 11; SDD 7; R 5	11 (47.8)
Voriconazole	BP	18	0.12–8	0.25	2	S 8; I 7; R 3	8 (44.4)
Itraconazole	ECV	23	0.12–2	0.25	0.5	WT 21; NWT 2	21 (91.3)
Posaconazole	ECV	23	0.12–2	0.25	1	NWT 17; WT 6	6 (26.1)
Amphotericin B	ECV	23	0.25–1	0.5	1	WT 23	23 (100.0)
Anidulafungin	BP	23	0.12–0.25	0.12	0.25	S 23	23 (100.0)
Micafungin	BP	23	0.015–0.12	0.03	0.12	S 23	23 (100.0)
Caspofungin	BP	23	0.06–0.5	0.25	0.5	S 20; I 3	20 (87.0)
Flucytosine	—	23	0.06–0.25	0.12	0.25	NI	—
***Candida parapsilosis* (n = 10)**							
Fluconazole	BP	10	0.5–4	0.5	4	S 6; SDD 4	6 (60.0)
Voriconazole	BP	10	0.008–0.06	0.015	0.06	S 10	10 (100.0)
Itraconazole	ECV	10	0.015–0.25	0.06	0.25	WT 10	10 (100.0)
Posaconazole	ECV	10	0.015–0.12	0.03	0.12	WT 10	10 (100.0)
Amphotericin B	ECV	10	0.12–0.5	0.25	0.5	WT 10	10 (100.0)
Anidulafungin	BP	10	0.25–4	2	2	S 9; I 1	9 (90.0)
Micafungin	BP	10	0.5–4	2	2	S 9; I 1	9 (90.0)
Caspofungin	BP	10	0.25–2	0.5	1	S 10	10 (100.0)
Flucytosine	—	10	0.06–64	0.06	64	NI	—
Candida albicans (n = 7)							
Fluconazole	BP	7	0.25–1	—	—	S 7	7 (100.0)
Voriconazole	BP	7	0.008–0.06	—	—	S 7	7 (100.0)
Itraconazole	ECV	7	0.015–0.12	—	—	WT 7	7 (100.0)
Posaconazole	ECV	7	0.015–0.06	—	—	WT 7	7 (100.0)
Amphotericin B	ECV	7	0.25–0.5	—	—	WT 7	7 (100.0)
Anidulafungin	BP	7	0.03–0.12	—	—	S 7	7 (100.0)
Micafungin	BP	7	0.008–0.015	—	—	S 7	7 (100.0)
Caspofungin	BP	7	0.06–0.12	—	—	S 7	7 (100.0)
Flucytosine	—	7	0.06–0.5	—	—	NI	—
***Candida pseudohaemulonii* (n = 3)**							
Fluconazole	—	3	1–2	—	—	NI	—
Voriconazole	—	3	0.015–0.06	—	—	NI	—
Itraconazole	—	3	0.015–0.12	—	—	NI	—
Posaconazole	—	3	0.008–0.03	—	—	NI	—

Amphotericin B	—	3	0.25–1	—	—	NI	—
Anidulafungin	—	3	0.03–0.12	—	—	NI	—
Micafungin	—	3	0.06–0.12	—	—	NI	—
Caspofungin	—	3	0.03–0.12	—	—	NI	—
Flucytosine	—	3	0.06–0.12	—	—	NI	—
***Candida orthopsilosis* (n = 1)**							
Fluconazole	—	1	1–1	—	—	NI	—
Voriconazole	—	1	0.03–0.03	—	—	NI	—
Itraconazole	—	1	0.12–0.12	—	—	NI	—
Posaconazole	—	1	0.06–0.06	—	—	NI	—
Amphotericin B	—	1	0.5–0.5	—	—	NI	—
Anidulafungin	—	1	0.12–0.12	—	—	NI	—
Micafungin	—	1	0.5–0.5	—	—	NI	—
Caspofungin	—	1	0.12–0.12	—	—	NI	—
Flucytosine	—	1	0.06–0.06	—	—	NI	—
***Candida guilliermondii* (n = 1)**							
Fluconazole	—	1	16–16	—	—	NI	—
Voriconazole	—	1	0.25–0.25	—	—	NI	—
Itraconazole	—	1	1–1	—	—	NI	—
Posaconazole	—	1	0.5–0.5	—	—	NI	—
Amphotericin B	—	1	1–1	—	—	NI	—
Anidulafungin	—	1	1–1	—	—	NI	—
Micafungin	—	1	0.5–0.5	—	—	NI	—
Caspofungin	—	1	0.5–0.5	—	—	NI	—
Flucytosine	—	1	0.06–0.06	—	—	NI	—

MIC, minimum inhibitory concentration; MIC50 / MIC90, MIC inhibiting 50% / 90% of isolates; BP, CLSI clinical breakpoint; ECV, epidemiological cutoff value; S, susceptible; SDD, susceptible-dose-dependent; I, intermediate; R, resistant; WT, wild-type; NWT, non-wild-type; NI, not interpretable.

Forty-five consecutive *Candida* bloodstream isolates from patients aged ≤18 years, tested 23 December 2021 – 7 May 2026. Species were identified by MALDI-TOF MS; MICs were determined with the Sensititre YeastOne YO10 panel, not the CLSI reference method.

Basis: BP, CLSI M27M44S-Ed3 (S, SDD, I, R); ECV, CLSI M57S-Ed4 (WT, NWT) — an ECV identifies acquired resistance and does not predict clinical response; NI, no criteria exist (flucytosine, and every agent for *C. pseudohaemulonii*, *C. orthopsilosis*, and *C. guilliermondii*), MIC shown for reference only.

Voriconazole was unreported for five *C. tropicalis* isolates (denominator 18). MIC50 / MIC90 should be interpreted with caution where fewer than 10 isolates were tested. Caspofungin results were corroborated against anidulafungin and micafungin. The single intermediate *C. parapsilosis* isolate reflects this species' intrinsically higher echinocandin MICs; *FKS1* sequencing was not performed, so acquired resistance cannot be formally excluded.

Table S2. Sites of *Candida* isolation among 117 children with proven invasive candidiasis

Site of *Candida* isolation	n (%) (N = 117)
Blood	110 (94.0)
Peritoneal fluid	16 (13.7)
Other sterile tissue or fluid*	9 (7.7)
Pleural fluid	4 (3.4)
Cerebrospinal fluid	2 (1.7)
Vitreous fluid	1 (0.9)
**Number of positive sterile sites per patient**	
1 site	96 (82.1)
2 sites	18 (15.4)
3 sites	2 (1.7)
4 sites	1 (0.9)

Denominator is all 117 patients; categories are not mutually exclusive, and 21 patients had Candida isolated from more than one site. * Other sterile tissue or fluid (9 patients) comprised deep tissue or fluid obtained under sterile technique, mostly intraoperatively (e.g., bone, deep tissue, and visceral biopsies). Three were catheter-associated specimens, which are not accepted as sterile-site cultures under EORTC/MSGERC criteria; however, all three patients had concurrent candidemia as the primary basis for diagnosis. In total, 6 of the 9 patients in this category also had candidemia.

Table S3. Antifungal agents administered to 105 treated children

Antifungal agent	n (%) of treated patients (n = 105)
Amphotericin B	96 (91.4)
Fluconazole	73 (69.5)
Echinocandin (micafungin or caspofungin)	15 (14.3)
Voriconazole	9 (8.6)
Flucytosine	5 (4.8)
Itraconazole	3 (2.9)
Posaconazole	0 (0.0)
**Number of antifungal agents received**	
1 agent	30 (28.6)
2 agents	59 (56.2)
3 agents	13 (12.4)
4 agents	2 (1.9)
5 agents	1 (1.0)

Denominator is the 105 patients who received targeted antifungal therapy; categories are not mutually exclusive. The number of agents per patient reflects sequential exposure over admission—as empiric therapy was switched to targeted therapy once species and susceptibility were known—rather than concurrent combination therapy. The one patient who received five agents illustrates this: a child with leukemia and prolonged neutropenia on itraconazole prophylaxis who developed breakthrough candidemia and was switched sequentially through fluconazole, amphotericin B, voriconazole, and finally micafungin plus voriconazole as complications and azole-resistant *C. tropicalis* emerged. No patient received posaconazole. The data instrument records micafungin and caspofungin as a single category, so these agents cannot be distinguished.

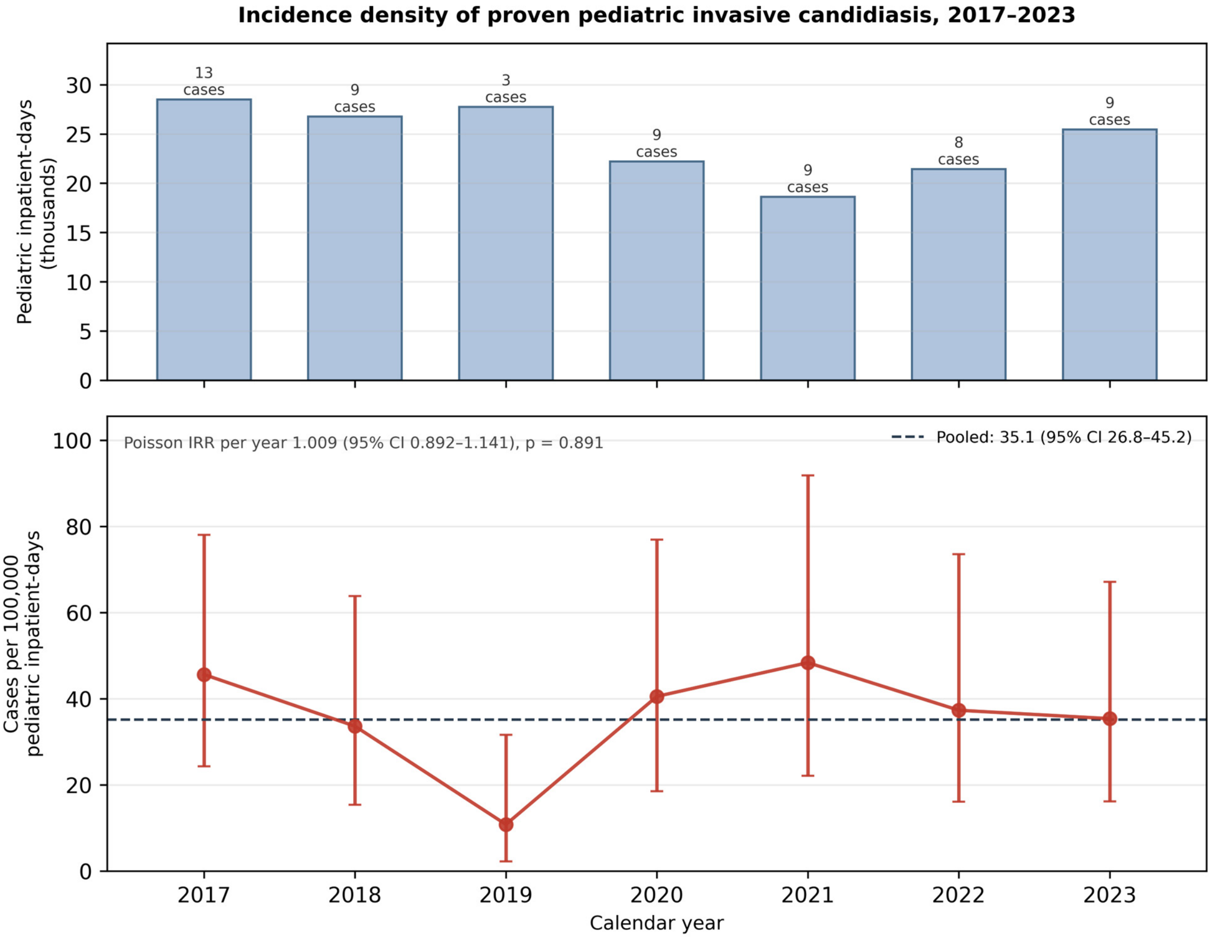

Denominator = total inpatient-days on pediatric wards, including NICU and PICU. Vertical bars are exact Poisson 95% confidence intervals.

Figure S1. Annual incidence density of pediatric invasive candidiasis, 2017–2023. Each point is the incidence density for that calendar year (proven IC cases per 100,000 pediatric inpatient-days), with vertical bars showing the exact Poisson 95% confidence interval; the denominator is total annual inpatient-days on the pediatric wards, including the neonatal and pediatric intensive care units. The pooled incidence density over the period was 35.1 per 100,000 inpatient-days (95% CI, 26.8–45.2). Poisson regression showed no temporal trend (incidence rate ratio 1.009 per year; 95% CI, 0.892–1.141; $p = 0.891$). The low case count in 2019 (n = 3) produces a wide interval and drives much of the year-to-year variation. Denominator data were available only from 2017, so earlier years are not shown.

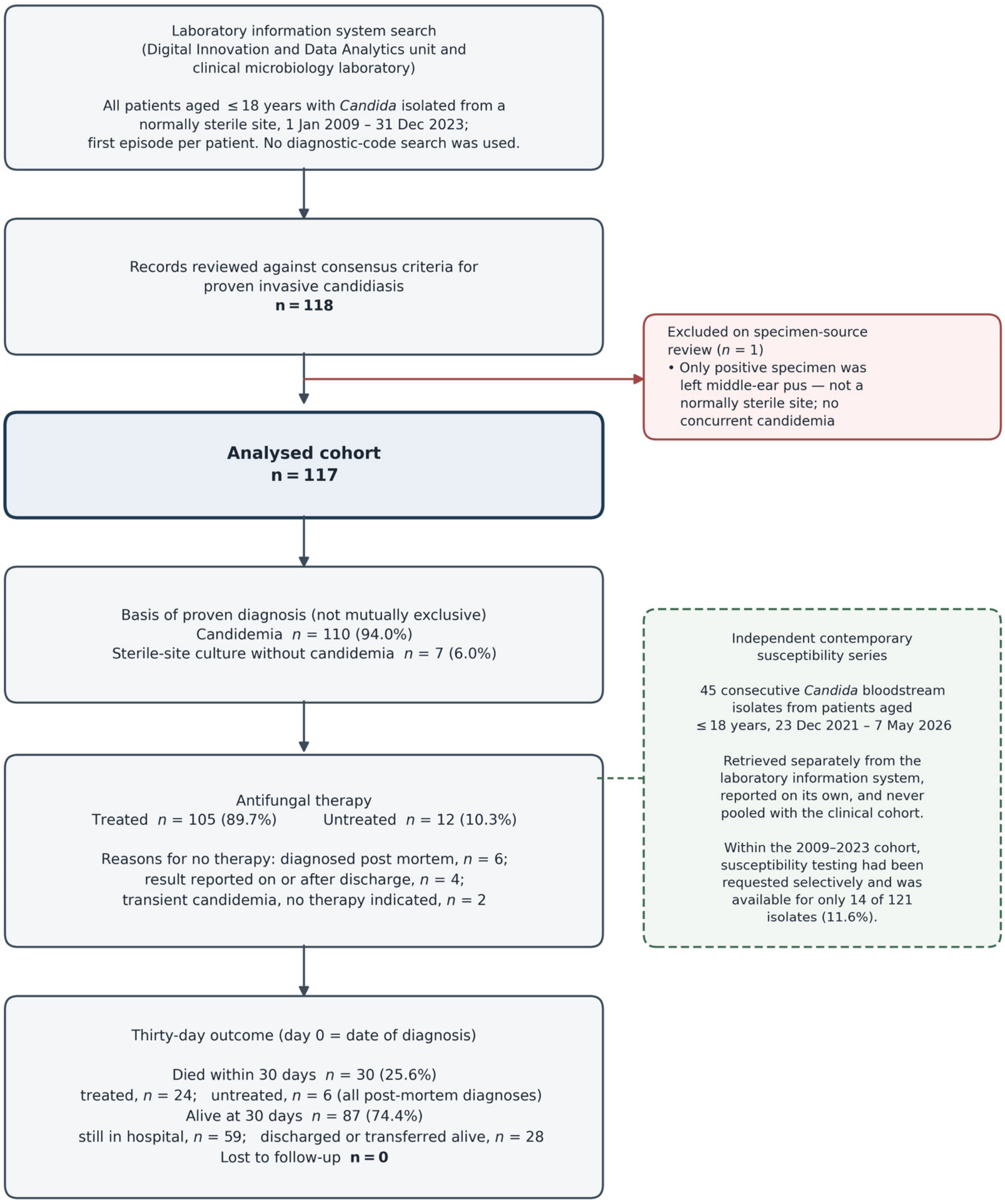

Figure S2. Flow of participants through the study. Case ascertainment was culture-based: all patients aged ≤18 years with *Candida* isolated from a normally sterile site during 2009–2023 were retrieved from the laboratory information system, restricted to the first episode per patient. Route to proven diagnosis is not mutually exclusive: seven children met the criteria on the basis of a sterile-site culture without concurrent candidemia. The contemporary antifungal susceptibility series (45 consecutive pediatric *Candida* bloodstream isolates, December 2021 – May 2026) was assembled independently from the laboratory information system and was never pooled with the clinical cohort; within the cohort itself, susceptibility testing had been requested selectively and was available for only 14 of 121 isolates.

File S1. STROBE Statement — Checklist of items that should be included in reports of *cohort studies*

Manuscript: Persistent Non-albicans Predominance, High Mortality, and Azole-Non-Susceptible Candida tropicalis: A 15-Year Pediatric Invasive Candidiasis Cohort in Southern Thailand

Locations refer to numbered sections of the manuscript. Page numbers should be inserted at proof stage.

	Item No.	Recommendation	Reported in
**Title and abstract**	1	(*a*) Indicate the study's design with a commonly used term in the title or the abstract	Title ("15-Year Retrospective Cohort")
		(*b*) Provide in the abstract an informative and balanced summary of what was done and what was found	Abstract
**Introduction**			
Background/rationale	2	Explain the scientific background and rationale for the investigation being reported	Section 1, paragraphs 1–3
Objectives	3	State specific objectives, including any prespecified hypotheses	Section 1, final paragraph
**Methods**			
Study design	4	Present key elements of study design early in the paper	Section 2.1, first sentence; Abstract
Setting	5	Describe the setting, locations, and relevant dates, including periods of recruitment, exposure, follow-up, and data collection	Section 2.1 (cohort, 1 Jan 2009 – 31 Dec 2023); Section 2.3 (susceptibility series, 23 Dec 2021 – 7 May 2026)
Participants	6	(*a*) Give the eligibility criteria, and the sources and methods of selection of participants. Describe methods of follow-up	Section 2.1 (culture-based ascertainment from the laboratory information system; proven-IC criteria; exclusions; first episode per patient). Follow-up: Section 3.3
		(*b*) For matched studies, give matching criteria and number of exposed and unexposed	Not applicable — unmatched cohort
Variables	7	Clearly define all outcomes, exposures, predictors, potential confounders, and effect modifiers. Give diagnostic criteria, if applicable	Section 2.1 (diagnostic criteria for proven IC); Section 2.2 (variable definitions, primary outcome); Section 2.4 (pre-specified covariates)
Data sources / measurement	8	For each variable of interest, give sources of data and details of methods of assessment	Section 2.1 (laboratory information system); Section 2.2 (standardized REDCap form from electronic medical records); Section 2.3 (species identification

	Item No.	Recommendation	Reported in
		(measurement). Describe comparability of assessment methods if there is more than one group	by MALDI-TOF MS; Sensititre YeastOne YO10 panel; CLSI M27M44S-Ed3 clinical breakpoints and CLSI M57S-Ed4 epidemiological cutoff values)
Bias	9	Describe any efforts to address potential sources of bias	Section 2.2 (date-consistency audit; retrieval of culture reporting dates); Section 2.4 ("no antifungal treatment" excluded before modeling because of reverse causation; competing-risks analysis for informative censoring; findings from selectively performed investigations reported with the number examined as denominator); Section 4, final two paragraphs
Study size	10	Explain how the study size was arrived at	Section 2.1 (all eligible patients in a fixed 15-year window; no sample-size calculation was performed); Section 4, final two paragraphs (modest number of events)
Quantitative variables	11	Explain how quantitative variables were handled in the analyses. If applicable, describe which groupings were chosen and why	Section 2.2 (thrombocytopenia defined as platelet count <150,000/μL; neonate defined as age <1 month); Section 2.4 (continuous variables summarized as median [IQR]); Section 2.4 (incidence density with calendar year as a continuous covariate)
Statistical methods	12	(*a*) Describe all statistical methods, including those used to control for confounding	Section 2.4, "Primary multivariable model" (Firth penalized logistic regression; profile penalized-likelihood CIs; penalized likelihood-ratio tests)
		(*b*) Describe any methods used to examine subgroups and interactions	Section 2.4 (stratified Kaplan–Meier analyses with log-rank tests); Section 2.4, "Temporal trends" (early vs late period; Cochran–Armitage trend across three periods). No interaction terms were fitted
		(*c*) Explain how missing data were addressed	Section 2.4 (no missing values among modeled covariates; structural missingness from conditional data entry not imputed, and the relevant findings reported with the number examined as denominator); Table S1 footnote (voriconazole results missing for five *C. tropicalis* isolates; no imputation; bounding analysis reported)
		(*d*) If applicable, explain how loss to follow-up was addressed	Section 2.4, "Time-to-event analyses" (patients discharged or transferred alive before day 30 censored at last known contact and additionally modeled as a competing event); Section 3.3 (no patient lost to follow-up)
		(*e*) Describe any sensitivity	Section 2.4, "Sensitivity analyses" (five pre-specified

	Item No.	Recommendation	Reported in
		analyses	analyses, model S1–S5); results in Table 3
**Results**			
Participants	13	(*a*) Report numbers of individuals at each stage of study	Figure S2; Sections 2.1 and 3.1: the laboratory-based search returned 118 patients; 1 was excluded after specimen-source review (middle-ear pus, non-sterile site); 117 analysed. All 117 completed 30-day follow-up or reached a terminal event
		(*b*) Give reasons for non-participation at each stage	Figure S2; Section 2.1 (exclusion criteria and the single exclusion, with its reason)
		(*c*) Consider use of a flow diagram	Figure S2
Descriptive data	14	(*a*) Give characteristics of study participants and information on exposures and potential confounders	Table 1; Section 3.1
		(*b*) Indicate number of participants with missing data for each variable of interest	Section 2.4 (no missing values for any modeled covariate, age, sex, or platelet count); Section 3.1 (denominators stated for selectively performed investigations: 52 imaged, 38 funduscopy, 33 echocardiography)
		(*c*) Summarise follow-up time (e.g., average and total amount)	Section 3.3 (30-day follow-up window with no loss to follow-up; 28 children discharged alive before day 30, handled by competing-risks analysis)
Outcome data	15	Report numbers of outcome events or summary measures over time	Section 3.3 (30 deaths; Kaplan–Meier and Aalen–Johansen estimates); Figure 1
Main results	16	(*a*) Give unadjusted estimates and, if applicable, confounder-adjusted estimates and their precision. Make clear which confounders were adjusted for and why they were included	Table 2 (crude and adjusted ORs with profile penalized-likelihood 95% CIs); Section 2.4 states the pre-specification rule for covariate selection; Section 3.4
		(*b*) Report category boundaries when continuous variables were categorized	Section 2.2 and Table 1 (thrombocytopenia <150,000/µL; neutropenia <1000 cells/µL; neonate <1 month; prematurity <37 weeks' gestation)
		(*c*) If relevant, consider translating estimates of relative risk into absolute risk for a meaningful time period	Section 3.3 reports absolute 30-day mortality overall and by stratum; Section 3.5 reports absolute incidence density per 100,000 inpatient-days
Other analyses	17	Report other analyses done — e.g., analyses of subgroups and interactions, and sensitivity	Table 3 (sensitivity analyses model S1–S5 and Fine–Gray model); Section 3.5 (incidence density and Poisson/negative-binomial trend); Section 3.6

	Item No.	Recommendation	Reported in
		analyses	(temporal trends and species-specific mortality, labelled exploratory); Section 3.7 and Table S1 (susceptibility series)
**Discussion**			
Key results	18	Summarise key results with reference to study objectives	Section 4, paragraphs 1–2; Section 5
Limitations	19	Discuss limitations of the study, taking into account sources of potential bias or imprecision. Discuss both direction and magnitude of any potential bias	Section 4, final two paragraphs (limitations given with the direction of the expected bias: reverse causation quantified in Table 3; selection and survivorship bias in the imaging subgroup quantified in Section 4; informative censoring addressed by the competing-risks estimate; non-random missingness of voriconazole results bounded at 34.8–56.5%)
Interpretation	20	Give a cautious overall interpretation of results considering objectives, limitations, multiplicity of analyses, results from similar studies, and other relevant evidence	Section 4 (comparison with regional and international cohorts; exploratory analyses explicitly labelled and unadjusted for multiplicity; published resistance mechanisms presented as plausible explanations rather than findings demonstrated in this cohort)
Generalisability	21	Discuss the generalisability (external validity) of the study results	Section 4, final two paragraphs (single-center retrospective design; referral bias)
**Other information**			
Funding	22	Give the source of funding and the role of the funders for the present study and, if applicable, for the original study on which the present article is based	Funding statement (back matter): no external funding; article processing charge funded in part by the Faculty of Medicine, Prince of Songkla University, and in part by the corresponding author, who is an author and therefore had a role in all aspects of the work

Note. An Explanation and Elaboration article discusses each checklist item and gives methodological background and published examples of transparent reporting. The STROBE checklist is best used in conjunction with this article (freely available on the websites of PLoS Medicine at http://www.plosmedicine.org/, Annals of Internal Medicine at http://www.annals.org/, and Epidemiology at http://www.epidem.com/). Information on the STROBE Initiative is available at http://www.strobe-statement.org.
